# Executive Functioning in 60+ Autistic Males: The Discrepancy Between Experienced Challenges and Cognitive Performance

**DOI:** 10.1007/s10803-020-04368-9

**Published:** 2020-01-17

**Authors:** Hilde M. Geurts, S. E. Pol, J. Lobbestael, Claudia J. P. Simons

**Affiliations:** 1 Dutch Autism & ADHD Research Center (d’Arc), Department of Psychology, University of Amsterdam, Postbus 15915, 1001 NK Amsterdam, The Netherlands; 2 Dr. Leo Kannerhuis (Youz/Parnassiagroup), Autism Clinic, Amstedam, The Netherlands; 3 GGzE, Institute for Mental Health Care Eindhoven and De Kempen, Centre for Elderly Psychiatry, Eindhoven, The Netherlands; 4 Department of Clinical Psychological Science, Faculty of Psychology and Neuroscience, Maastricht University, Maastricht, The Netherlands; 5 Maastricht University Medical Center, Department of Psychiatry and Neuropsychology, School for Mental Health and Neuroscience, Maastricht University, Maastricht, The Netherlands; 6 Present Address: Reinier Van Arkel Group, ’s Hertogenbosch, The Netherlands

**Keywords:** Old age, Autism, Cognition, Executive function

## Abstract

As executive functioning (EF) is especially sensitive to age-related cognitive decline, EF was evaluated by using a multi-method assessment. Fifty males (60–85 years) with a late adulthood autism spectrum condition (ASC) diagnosis and 51 non-ASC males (60–83 years) were compared on cognitive tests across EF domains (cognitive flexibility, planning, processing speed, and working memory) and a self- and proxy report of the Behavior Rating Inventory of Executive Function-Adult Version. While no objective performance differences emerged, autistic males and their proxies did report more EF challenges than non-ASC males on the subjective measure. In order to know how to support the older autistic men who received their ASC diagnosis in late adulthood with their daily life EF challenges, it is important to understand what underlies these subjective EF problems.

Aging in adulthood is typically associated with cognitive decline. One cognitive domain that seems especially sensitive to age-related cognitive decline is executive functioning (EF; e.g., Diamond 2013). EF is traditionally used as an umbrella term for functions such as working memory (WM), impulse control, inhibition, planning, and cognitive flexibility (e.g., Diamond 2013). EF problems are not only common among older people. Children, adolescents, and young adults with an autism spectrum condition (ASC) diagnosis are known to encounter EF problems across various EF domains (e.g., Demetriou et al. 2018; Pennington and Ozonoff 1996; Wallace et al. 2016). In the past few years, interest in cognitive aging of people with an ASC has increased, but studies assessing EF in this specific sample are still scarce. We will, therefore, focus on EF in autistic adults over 60 years of age.

To our knowledge only seven papers reported on objective clinical EF measures of a (clinically) diagnosed ASC adult sample of which approximately 50% of the participants was over 45 years of age (Braden et al. 2017; Davids et al. 2016; Geurts and Vissers 2012; Lever and Geurts 2016a; Powell et al. 2017; Tse et al. 2019; Walsh et al. 2019). The findings, however, are inconsistent. In a first small study (Geurts and Vissers 2012; N_ASC_ = 23, N_non-ASC_ = 23, age 51–83 years), next to episodic memory and general processing speed measures, the EFs planning, cognitive flexibility, inhibition, WM, and generativity (i.e., verbal fluency) were assessed. Results showed that the ASC group performed worse than the non-ASC group on WM and generativity, but not on any of the other EF measures. Exploratory analyses suggested that, depending on the cognitive domain, those with ASC showed larger age related-differences (non-EF domain visual episodic memory) and reduced age-related differences (generativity), but predominantly similar age-related differences (all other cognitive domains). This was the first study to report that age may disproportionally affect specific cognitive functions in autistic adults. Moreover, the ASC participants did report much more cognitive difficulties on a subjective measure as compared to those without ASC (same participants, different paper: van Heijst and Geurts 2015). In a similar study (Davids et al. 2016; N_ASC_ = 36, N_non-ASC_ = 36, age 50–84 years), focusing on some of the same cognitive domains, no large differences in performance between the ASC and non-ASC group were observed in neither general processing speed nor planning (although the ASC group was slower) and generativity. Interestingly, ASC participants did report many EF problems in daily life. Moreover, their proxies also observed that the autistic adults experienced daily life EF problems. In yet another recent and similar study (Tse et al. 2019; N_ASC_ = 28, N_non-ASC_ = 27, age 50–72 years) the ASC group was generally slower and performed less well on visual WM as compared to the non-ASC group. Unfortunately, in these studies age-related differences were not explored. As age-range, IQ range, gender balance, and age-of-diagnoses were rather similar across these studies, these factors were not likely to explain the differences in findings with the Geurts and Vissers (2012) study. But it could well be that in each of these three studies, some of the findings, may have been due to a type I error.

There were, however, also some differences in the cognitive measures used which might account for the discrepancy in findings. In two other small studies [Braden et al. 2017; N_ASC_ = 14 to 16, N_non-ASC_ = 17, age 40–64 years; Walsh et al. 2019; N_ASC_ = 24 to 25, N_non-ASC_ = 15 to 21, age 18 to 70 years (note, they included specific subgroups aged 40–70 with mean age over 45 years)] with slightly younger participants, EF differences emerged on a cognitive flexibility task similar to the one used in Geurts and Vissers (2012) study,^1^ but not on a planning task which was similar to the one used in the Davids et al. (2016) study. So even when similar tasks were used, the findings in across the different small studies are still inconsistent. Hence, replication in a larger sample is of importance.

When including again primarily adults with a late adulthood ASC diagnosis, but now focussing on a much wider age range (N_ASC_ = 118 to, N_non-ASC_ = 118–167 to; 19–79 years; see for detailed findings Lever and Geurts 2016a^2^; Lever et al. 2015, 2017) both the larger amount of self-reported cognitive difficulties (similar to Davids et al. 2016; van Heijst and Geurts 2015) and EF group differences in generativity (similar to Geurts and Vissers 2012) were replicated. However, no differences emerged between those older adults with and without ASC when using a different WM task (Lever et al. 2015, see also Braden et al. 2017). Furthermore, reduced age-related differences were observed for WM, but not for generativity which is in contrast to the 2012 Geurts and Vissers study. Regarding the relationship of WM with age, exploratory analyses revealed that IQ might have played a role. For those participants with a lower IQ (80–94), age-related differences were not apparent, while this was the case for those with a higher IQ (94–155). This was irrespective of having an ASC diagnosis or not. Also, findings from a recent small study including ASC adults across a wide age range (Powell et al. 2017; N_ASC_ = 29, N_non-ASC_ = 30, age 30–67 years) suggest that IQ might be of relevance. When including IQ as a covariate, similar age-related differences emerged for processing speed and verbal episodic memory. But, while for category learning reduced age-related differences (i.e., smaller group differences when older) were noted, for cognitive flexibility increased age-related differences (i.e., larger group differences when older) were reported. So, like in the 2012 Geurts and Vissers paper, Powell et al. (2017) show that in autistic adults’ age may disproportionally affect specific cognitive functions while other cognitive functions are unaffected. However, while Powell et al. (2017) argue that complex cognitive functions are mainly at risk for this accelerated aging effect, this hypothesis does not align with the findings of any of the previous studies. The inconsistency in the observed age-related patterns could be due to the chosen statistical analyses, but as there were many other methodological differences (age-range, IQ-range, type of EF tasks used, age of ASC diagnosis) it is hard to determine which factor(s) caused the observed difference in findings. As in the majority of included participants in each of the aforementioned studies received an ASC diagnosis in (late) adulthood (see also Abbott et al. 2018) age-of-diagnosis seems not to be a likely explaining factor for the inconsistency in findings.

In sum, the relationship between age and EF problems in old age ASC still remains unclear, due to contradictory neuropsychological results and overall small sample sizes. There is an insufficient focus on older adults as in most studies the mean age does not exceed 60 (except for Geurts and Vissers 2012; Davids et al. 2016; Tse et al. 2019), whilst it is known that various cognitive problems in healthy aging do not start to emerge until the age of 60 (Nilsson et al. 2009; Treitz et al. 2007). Therefore, in the current study we will focus a similar age-range and use similar EF tasks as in Geurts and Vissers (2012) and Davids et al. (2016). Moreover, like in these previous studies, the included participants will all be adults with a late adulthood diagnosis of ASC. This enables us to test which of the reported age-related differences in these complex cognitive functions (WM, cognitive flexibility, planning) will (or will not) be replicated. Moreover, we will determine how IQ might have an impact on the pattern of findings. Next to the objective measures, we will include a subjective EF measure to test whether we can replicate the increased report of general EF problems by ASC individuals themselves and by their proxies (Davids et al. 2016; Wallace et al. 2016).

## Methods

### Participants

In total 101 males^3^ aged 60 to 85 were included in this study. Individuals with an ASC diagnosis (n = 50) were recruited via a mental health institution in the Netherlands specialized in old age psychiatry and ASCs. All of them were referred to this specialist ASC team for a first diagnosis of ASC after which they were immediately included in the current study (see for a similar approach Abbott et al. 2018). We applied the following inclusion criteria: (a) age above 60; (b) a clinical ASC late adulthood diagnosis by the specialist ASC team (according to the DSM-IV American Psychiatric Association 2000); (c) IQ higher or equal to 80^4^ as established with a full IQ test; (d) no active infection, known genetic abnormalities, metabolic disorder, tuberculosis, epilepsy, or traumatic brain injury; (e) no disturbance of consciousness, delirium, psychosis, suicidal tendencies, severe aphasia, or major sensorimotor impairment precluding neuropsychological testing; (f) no substance abuse. Criteria (d)–(f) were based on available medical data, clinical observation, and a clinical interview by a trained ASC professional and as discussed by the multidisciplinary team including a medical doctor and a clinical psychologist. The participants for the non-ASC comparison (COM) group were recruited through advertisements in local newspapers, within the researchers’ social environment, and via several organizations where healthy older adults are known to volunteer. All participants were male. For age and IQ individuals were selected to fit within the same age- and IQ range of the ASC group, but they were not individually matched. The following inclusion criteria were applied: (a) age above 60; (b) no self-reported ASC, or any other current psychiatric diagnosis; (c) no (self-reported) first degree family members with ASC; (d) IQ > 80. Both (c) and (d) were verified with a short interview by the psychologist. For the detailed characteristics regarding age, IQ, and educational level of the sample, see Table 1. The ASC group was slightly younger than the COM group, but there were no differences with respect to IQ or education.

**Table 1:** Sample characteristics

	Group		Statistics
	ASC (n = 50)	COM (n = 51)	
	M (SD; range)	M (SD; range)	
Age^a^	65.8 (5.6; 60–85)	69.7 (5.6; 60–83)	*t*(99) = − 3.30, *p* = .001
AQ^b^	123.5 (33.2; 18–172)	NA	*–*
TIQ	110.7 (12.2; 79–139)	110.7 (12.5; 83–139)	*t*(99) = − .02, *p* = .987
Education^c^	0/1/1/7/11/22/8	0/0/0/2/12/28/9	*χ*^2^(5) = 5.59, *p* = .348

*ASC* autism spectrum condition, *AQ* Autism Spectrum Questionnaire total score, *COM* comparison group (non-ASC group), *TIQ* total IQ

^a^The age of diagnosis is in this study the same as age when included, as autistic adults were included in the study when they just received their clinical diagnosis

^b^The AQ was only administered by a subsample of the ASC group (N = 39)

^c^Education is a value between 1 and 7 as defined by Verhage (1964), the higher the number the higher someone’s education

Please note that proxies of all participants were also part of the current study. The participants themselves determined who they felt could give the most adequate information regarding their daily life. Moreover, the criterion to be considered a proxy was that the proxy needed to be the person who, in the past 5 years, had seen the autistic adult most often. The majority of the proxies were partners or sometimes children of the participants. However, for a few autistic participants, a clinical professional was considered to be the most appropriate proxy.

### Materials

#### Autism Assessment

There is no golden standard when diagnosing ASC in intellectually able older adults. Therefore, in addition to the clinical expertise of the professionals working at the specialist health care institute, we largely followed the Dutch multi-disciplinary guideline for assessing ASC in adults (Kan 2013). The ASC diagnosis was based on an unpublished standard diagnostic interview using the DSM-IV-TR criteria for ASC (precursor of the DSM-5 autism interview of Spek 2015), an extensive interview with a significant other (same semi-structured interview), clinical observations, and, if administered, the Autism-Spectrum Quotient (AQ; Baron-Cohen et al. 2001). The AQ is a valid and reliable self-report questionnaire for the assessment of autistic traits consisting of 50 items (see Table 1 for the AQ scores of the ASC group). A qualified clinician of our clinical centre, with at least 5 years’ experience in assessing older adults with ASC, combined the results from the interviews, observation, and questionnaires and conferred with the ASC expert team (consisting of two senior psychologists, a psychiatrist and a psychiatric nurse) before giving the definitive diagnosis.

#### Objective EF Assessment: Neuropsychological Tests

Next to the general intelligence test (full Wechsler Adult Intelligence Scale-Fourth Edition; WAIS-IV; Wechsler 2008; Dutch version 2014), we administered a total of three neuropsychological tests. For each of the tasks we chose the dependent measures that are most commonly used in the ASC and aging literature and were related to important aspects of EF: planning, cognitive flexibility, processing speed, and WM.

*Planning* two tasks, also used in the Davids et al. (2016) study, were included: the Delis–Kaplan Executive Function Systems Tower Task (DKEFS Tower; Delis et al. 2004) and Zoo Map task of the Behavioural Assessment of the Dysexecutive Syndrome (BADS Zoo Map; Wilson et al. 1998). The DKEFS Tower requires the participant to move five disks of varying size across three pegs so that disks are moved onto a designated peg and stacked according to size in the least number of moves possible. Both total time to complete the task and total score are included as dependent measures. The BADS Zoo Map consists of two parts. Part 1 requires the participant to plan a solution to a problem that requires the consideration of a number of rules: visiting only certain animals and places in a zoo, keeping to the paths and walking along certain paths only once. Points are awarded for visiting the animals in an optimal sequence or are subtracted for breaking any of the rules. Part 2 requires the participant to follow a set of instructions while following some simple rules; here the participant repeats the task but is provided with the order in which to visit the animals, removing most of the planning requirements. The difference in points between Parts 1 and 2 results in a so called profile score, which ranges from 0 to 4. Higher profile scores represent better planning. This profile score was used as dependent measure.

*Cognitive flexibility* the digital version of the Wisconsin Card Sorting Task (WCST) was used (Berg 1948; the paper and pencil version was used in Braden et al. 2017; the abbreviated version in Geurts and Vissers 2012). The WCST consists of four stimulus cards that vary along three dimensions (colour, shape, and number). Participants are given 128 cards that vary along the same dimensions and are asked to match the cards in the deck with 1 of the 4 stimulus cards. The program tells participants if they have placed a card correctly or incorrectly, but does not reveal the sorting strategy, which must be inferred from the provided feedback. Once 10 consecutive cards have been categorized correctly, the sorting principle changes without warning or comment. All sorts made according to the previous strategy now receive negative feedback. Participants are then expected to use the computer’s feedback to shift to a new categorization principle. The test continues until 6 categories have been correctly sorted or until all 128 cards have been used. The most widely used and sensitive variable is the number of perseverations (i.e., continued sorting by a previously correct principle despite feedback to the contrary; see Sergeant et al. 2002). Therefore, the number of perseverative responses was used as the dependent measure.

*General IQ*, *processing speed*, *and WM* the WAIS-IV (Wechsler 2008) is a test of general intellectual ability comprising subtests spanning four domains of cognitive functioning, namely, verbal comprehension, perceptual reasoning, WM, and processing speed. The overall score on this test will be used to measure general IQ (TIQ), as this is needed to check the inclusion criteria. Like in Davids et al. (2016) the processing speed index (PSI) is used as part of the EF assessment (see also Tse et al. 2019). We also included the WM index (WMI) to cover the WM domain. The core WMI subtests are Digit Span and Arithmetic. Digit Span includes three tasks: Forward, Backward, and Sequencing. For the Forward task, the participant repeats numbers spoken by the examiner. The Backward task requires the participant to repeat those numbers in reverse order, and the Sequencing task requires the participant to sequence numbers from the lowest to the highest number. There is no time limit for the participant to respond, but the examiner reads each number out aloud at the rate of one number per second. Arithmetic requires the participant to mentally solve arithmetical word problems within a time limit. The core PSI subtests include Symbol Search and Coding. Symbol Search requires the participant to search for two target symbols within a row of symbols. Participants use a pencil to mark either the matching symbol or a “no” box to indicate responses, and have 120 s to complete as many rows (items) as possible. Coding requires that the participant copies simple symbols as quickly as possible, based on a key that pairs numbers with the symbols and is again given 120 s to complete the subtest. Both WMI and PSI are the dependent measures.

#### Subjective EF Assessment: Self-report and Proxy-Report Questionnaire

The Behavior Rating Inventory of Executive Function-Adult version (BRIEF; Roth et al. 2005) was used in the study of Davids et al. (2016) and has demonstrated sensitivity to EF strengths and challenges in various clinical populations (Rabin et al. 2006). It is a self- and proxy report measure developed to assess the everyday behavioural manifestations of adults’ (aged 18–90 years) executive functions (Roth et al. 2005). The BRIEF consists of equivalent Self-Report and Proxy Report Forms, each having 75 items in 9 non-overlapping scales, as well as 2 summary index scales and one scale reflecting overall functioning (Global Executive Composite). The questions are rated on a three-point Likert scale (never, sometimes and often). Example questions are; ‘I find it difficult to make decisions’ or ‘I am having trouble organising’. Because we are interested in a general indication of perceived EF problems and in order to reduce the number of statistical comparisons, we will solely focus on the Global Executive Composite score (BRIEF total score) for both self- and proxy reports. Please note that the proxies were chosen by the participants as we asked them who they believed knew them the best. They needed to know each other for at least 5 years.

### Procedure

Participants were informed about the study purposes and procedure and both verbal and written informed consent was obtained. The ASC group was tested in three sessions, in which the ASC diagnosis was confirmed and neuropsychological tests were administered. The COM participants were all interviewed briefly in order to check the study inclusion criteria. Next neuropsychological tests were administered in one session. Similar to the study of Davids et al. (2016), neuropsychological tests were administered in the same order in both groups (demographic questionnaire, WAIS-IV, DKEFS-tower, BADS Zoo Map, WCST, and BRIEF). The session took between 170 and 195 min. All participants were debriefed and a reimbursement for travel expenses was offered. The proxy questionnaires were filled out at home and send to the researchers via regular mail. Data was collected between June 2014 and August 2015. The study was approved by a Medical and Ethical Board (METC Brabant: NL45575.008.13).

### Statistical Analyses

For the data analyses SPSS IBM 24 statistical software (IBM 2016) as well as JASP version 9.0 (JASP Team 2018) and StatCheck (Epskamp and Nuijten 2016) were used. For the continuous descriptive measures (age, IQ) and for the ordinal measures (education), respectively t-tests and a Chi-square test were used. Checking normality (Shapiro–Wilk) and the outliers (scores outside the 3 * interquartile range) revealed neither significant deviations from normality or outliers in any of the objective and subjective EF measures. Therefore, we first compared the ASC group and the COM on the objective and subjective EF measures with five (M)ANOVAs with group as between subject factor so the current findings can be compared to both Geurts and Vissers (2012) and Davids et al. (2016). For all measures, except WMI and PSI, the analyses were rerun with IQ as covariate. A Bonferroni correction was used for the analyses with and without covariate to correct for the number of analyses (ANOVA α 0.01; ANCOVA 0.013). However, the main analyses are the regression analyses were the predictor variables included group (ASC/COM), age (mean-centred), and the age by group interaction term similar to Geurts and Vissers (2012) and Lever and Geurts (2016a, b). To explore whether the pattern of findings change when IQ was taken into account, the regression analyses were rerun with IQ (i.e., please note that WMI and PSI are now excluded as the dependent measures) entered in the first step of the regression analyses (see for a similar approach Powell et al. 2017). Pearson correlations (one-tailed) were calculated for exploratory purposes between (1) self- and proxy BRIEF-A and (2) subjective and objective measurements.

Additionally we performed Bayesian analyses to assess the strength of evidence for the findings (Jeffreys 1961) for the group comparisons. The prior was set at 0.05 for the fixed effects, following the JASP version 0.9 standard. Bayesian hypothesis testing quantifies the extent to which the data support an alternative hypothesis H_1_ against the null hypothesis H_0_, as expressed by the Bayes factor (Rouder et al. 2009), BF_10_. A Bayes factor of 1 or lower indicates no evidence for the alternative hypothesis over the null hypothesis (i.e., no difference), 1–3 anecdotal, 3–10 moderate, 10–30 strong, 30–100 very strong, and > 100 extreme evidence for the alternative hypothesis (Wagenmakers et al. 2011). We will also report BF_01_ (note that BF_01_ = 1/BF_10_), which is the Bayes factor indicating whether there is (more) evidence for the null hypothesis over the alternative hypothesis. So when, for example, BF_01_ = 2 (i.e., anecdotal evidence), it is two times more likely to observe the pattern of findings under the assumption that there is no difference between the groups as opposed to assuming that there is an actual performance difference between the groups.

## Results

### Objective EF Assessment: Neuropsychological Tests

There were no statistically significant differences between groups in any of the objective cognitive measures (see Table 2). The Bayes factors showed that there was no evidence for the hypothesis that there is a group difference when compared to the alternative of a lack of a group difference (see Table 2 for BF_10_ values which were all below 1). Moreover, for many measures, there was only anecdotal evidence for the lack of a difference between the groups compared to the alternative hypothesis that there was an actual difference (see Table 2 for BF_01_ values which were between 1 and 3 for DKEFS total score, WCST perseverative responses, and WMI). For the remaining measures (BADS Zoo Map, DKEFS total time, PSI), there was moderate evidence that there was indeed no difference in performance between the ASC and COM group. This combination of findings implies that when there is an actual group difference, it is likely to be a rather small effect, which is in line with the observed small effect sizes (see Table 2). Covarying for IQ did not alter the pattern of findings. As the ASC and COM group differed in age, and age might impact the group findings, age could be considered as covariate to test the impact of age on the observed group findings. Even though the impact of age was considered to be tested the planned regression analyses, we reran the (M)ANOVAs with age as covariate. This only resulted in a significant groups difference on the DKEFS total score (*p* = 0.19). However, the Bayes factor still showed that there was no evidence for the hypothesis that there is a group difference when compared to the alternative of a lack of a group difference (BF_10_ = 0.72).

**Table 2:** Group means, standard deviations and statistics for the objective and subjective executive functioning measures

Domain	Task	Dependent variable	Group		Statistics^d^			
			ASC M (SD; range)	COM M (SD; range)	*F*	*η*_p_^2^	BF_10_	BF_01_
Planning	BADS	Zoo profile score	2.5 (1.3; 0–4)	2.6 (1.1; 0–4)	< 1	.00	0.22	4.59
	DKEFS^a^	Tower total time	553.6 (178.6; 181–861)	567.2 (134.1; 324–859)	< 1	.00	0.23	4.38
		Tower performance	17.0 (3.6; 10–26)	18.2 (3.4; 12–26)	2.8	.03	0.72	1.39
Flexibility	WCST	Perseverative resp.	12.3 (8.4; 2–41)	15.0 (10.7; 1–52)	2.0	.02	0.51	1.96
WM	WAIS^b^	Index score	107.9 (15.3; 65–146)	111.1 (13.7; 86–143)	1.2	.01	0.36	2.76
PS		Index score	103.6 (16.6; 76–152)	102.8 (13.3; 84–145)	< 1	.00	0.22	4.59
EF	BRIEF^c^	Total score self	132.4 (22.1; 85–190)	100.0 (16.4; 73–138)	70.3*	.42	1.58e+10	6.32e−11
		Total score proxy	142.0 (24.6; 83–194)	101.1 (20.1; 70–155)	85.8*	.46	1.07e+12	9.33e−13

*ASC* autism spectrum condition, *BADS* Behavioural Assessment of the Dysexecutive Syndrome Zoo Map test, *COM* comparison, *DKEFS* Delis–Kaplan Executive Function Systems Tower Task, *BRIEF* The Behavior Rating Inventory of Executive Function-Adult version, *PS* processing speed, *resp.* responses, *WAIS* Wechsler Adult Intelligence Scale-Fourth Edition, *WCST* Wisconsin Card Sorting Test, *WM* working memory

*p < .001

^a^MANOVA DKEFS: *F*(2, 98) = 3.3, *p* = .04, *η*_p_^2^ = .06. When covarying for IQ: IQ *F*(2, 97) = 11.0, *p* < .001, *η*_p_^2^ = .19; Group *F*(2, 97) = 3.3, *p* = .04, *η*_p_^2^ = .06

^b^MANOVA WAIS: *F*(2, 98) = 1.2, *p* = .31, *η*_p_^2^ = .02. Please note that as these measures are part of the TIQ measure, analyses were not rerun with IQ as covariate

^c^MANOVA BRIEF: *F*(2, 98) = 52.2, *p* < .001, *η*_p_^2^ = .52. When covarying for IQ: IQ *F*(2, 97) = 1.3, *p* = .29, *η*_p_^2^ = .03; Group *F*(2, 97) = 52.9, *p* < .001, *η*_p_^2^ = .52

^d^When covarying for IQ the pattern of findings did not alter. Please note IQ was not a covariate for WM and PS

The regression analyses revealed that there were no main effect of age and no interaction effects of group with age for any of the dependent measures (see Table 3). So when age was held as a constant at its mean, group was still no predictor of value. This suggests that age-related differences between the two groups, if any, are constant across age. Including IQ as additional predictor did not alter the main pattern of findings, but IQ itself was an important predictor for both the performance on the BADS and the DKEFS.

**Table 3:** Standardized β coefficients and p values of the regression models with Age, Group, and Age by Group as factors (with and without IQ)

	BADS		DKEFS				WCST		WAIS				BRIEF			
	Zoo score^a^		Time^b^		Performance^c^		Perseverations^d^		WMI^e^		PSI^f^		Self^g^		Other^h^	
	*β*	*p*	*β*	*p*	*β*	*p*	*β*	*p*	*β*	*p*	*β*	*p*	*β*	*p*	*β*	*p*
Group	− .03	.78	− .04	.66	− .17	.09	− .14	.15	− .11	.27	.03	.77	.64	.00	.68	.00
Age	− .07	.63	.16	.26	− .19	.18	.24	.09	.06	.69	.11	.47	− .04	.69	.03	.76
Age * Group	− .04	.80	.13	.37	− .07	.61	− .04	.79	.15	.28	− .02	.88	.03	.79	.10	.32
IQ Age * Group^A^	− .07	.62	.16	.21	− .10	.42	− .04	.79	NA	NA	NA	NA	.03	.79	.10	.32

*BADS* Behavioural Assessment of the Dysexecutive Syndrome Zoo Map test, *DKEFS* Delis–Kaplan Executive Function Systems Tower Task, *BRIEF* The Behavior Rating Inventory of Executive Function-Adult version, *NA* not available, *WAIS* Wechsler Adult Intelligence Scale-Fourth Edition, *WMI* working memory index, *PSI* processing speed index, *WCST* Wisconsin Card Sorting Test

^a^*R*^2^ = .01, *F*(3, 97) < 1, *p* = .79

^b^*R*^2^ = .07, *F*(3, 97) = 2.5, *p* = .07

^c^*R*^2^ = .09, *F*(3, 97) = 2.9, *p* = .04

^d^*R*^2^ = .06, *F*(3, 97) = 2.2, *p* = .09

^e^*R*^2^ = .05, *F*(3, 97) = 1.7, *p* = .17

^f^*R*^2^ = .01, *F*(3, 97) < 1, *p* = .83

^g^*R*^2^ = .42, *F*(3, 97) = 23.0, *p* < .001

^h^*R*^2^ = .46, *F*(3, 97) = 29.9, *p* < .001

^A^Age was centered in these analyses. Here the findings are reported for the Age by Group interaction when IQ was taken into account. IQ had a significant main effect for the dependent measures of the BADS and DKEFS. Information regarding the other statistics can be obtained via the first author

### Subjective EF Assessment: Self-report and Proxy-Report Questionnaire

When looking at the BRIEF total score for both the self- and proxy-report, the ASC group (and their respective proxies) reported more EF problems than the COM group. The evidence for the hypothesis that there is an actual difference between the groups in experienced daily life EF problems was extremely strong (BF_10_ > 100), which fitted well with the observed effect sizes in the group comparisons. Like with the subjective measures, there were no interaction effects of group with age for both BRIEF measures.

### Correlation Between Objective and Subjective Measurements

When exploring the correlations between the various neuropsychological measures and the BRIEF-A self-report and proxy-report, we only observed statistically significant small negative correlations between BRIEF and the DKEFS total performance score (see Table 4). For all other measures there were no significant correlations, implying that objective and subjective measurements were in general not or only a little bit associated. There was a strong statistically significant positive correlation of the self- and proxy report of the BRIEF. When exploring the correlations for each of the groups separately, the correlation for the ASC group was small to medium (0.38) and in the COM group medium to large (0.66).

**Table 4:** Correlations between objective and subjective executive function measures

Measures	BRIEF	
	Self-report	Proxy-report
BADS Zoo Map score	− .10	− .10
DKEFS Time	.06	.08
DKEFS Score	− .17	− .21
WCST Perseverations	− .07	− .01
WMI	− .09	− .12
PSI	− .03	.10
BRIEF Self	–	.71*
BRIEF Proxy	.71*	–

*N* = 101 for all analyses. Pearson correlations for BRIEF Self and Proxy were also calculated per group, ASC *r* = .38 and COM *r* = .66. Please note that the proxies were in general the participants’ partners or sometimes their child, but for a few autistic participants the clinical professional was the proxy

*BADS* Behavioural Assessment of the Dysexecutive Syndrome, *DKEFS* Delis–Kaplan Executive Function Systems, *WCST* Wisconsin Card Sorting Task, *WMI* working memory index, *PSI* processing speed index, *BRIEF* Behavior Rating Inventory of Executive Function-Adult version

*p < .001 one-tailed

## Discussion

In this study focussing solely on old autistic men (i.e., over 60 years of age, and with a late adulthood ASC diagnosis), the goal was (a) to assess EF by using a multi-method assessment of both objective and subjective measures and (b) to determine whether or not previous findings (Davids et al. 2016; Geurts and Vissers 2012; van Heijst and Geurts 2014) could be replicated. In none of the EF domains (including planning, cognitive flexibility, WM, and processing speed), performance differences on the neuropsychological tests were shown between older autistic males and their non-autistic counterparts. This calls into question whether objectifiable EF problems are a key characteristic of older autistic men with a late adulthood ASC diagnosis. However, as a group, the autistic men did report experiencing more EF problems in daily life as compared to the non-autistic group. The proxies of the autistic adults also noticed more daily life EF problems in the autistic adults. Moreover, age did not seem to have a differential impact on those with or without an ASC diagnosis, which is suggestive of a similar (i.e., parallel) aging pattern across both the objective and the subjective measures. None of these patterns of findings were impacted by IQ. The majority of our current findings mirror the findings of the highly similar study of Davids et al. (2016). Thus, in older autistic men with a late adulthood ASC diagnosis and (above) average IQ, EF problems in daily life are clearly reported on, while this is not reflected in their cognitive test performance and is not differentially impacted by their age.

The consistency of findings across the current study and the Davids et al. (2016) study, is in sharp contrast with the lack of consistency across the broader range of studies including older autistic adults (Braden et al. 2017; Geurts and Vissers 2012; Lever and Geurts 2016a, b; Powell et al. 2017; Tse et al. 2019; Walsh et al. 2019). This inconsistency in findings could be due to differences in sample characteristics (age, sex, IQ level), type of cognitive tasks included, chosen statistical analyses, and how intelligence levels were accounted for. Alternatively, it might also be the results of the fact that the majority of the studies (Braden et al. 2017; Geurts and Vissers 2012; Powell et al. 2017; Tse et al. 2019; Walsh et al. 2019) are relatively small and likely underpowered for the type of analyses run. While the current study is slightly larger, it could be argued it is still rather small. However, the novel addition of Bayesian analyses revealed that there is no clear evidence for the hypotheses that there is a performance difference between autistic adults and non-autistic adults. This suggests that if a difference is observed, it is likely to be rather small or even false.

Heterogeneity in cognitive profiles has been observed in (young) autistic adults (e.g., Hill and Bird 2006) and it is to be expected to be present in older autistic adults as well. Heterogeneity, in cognitive abilities, typically increases with age (e.g., Buczylowska and Petermann 2016). Therefore, it is likely that a subgroup of older autistic adults do show cognitive problems when tested, where the majority does perform well. In a future larger study, the presence of cognitive subgroups needs to be tested. For now the inconsistencies in the research literature on cognitive aging in autistic adults mainly teaches us ones more, that one needs to be careful when drawing conclusions when samples are small.

The lack of a relation between EF test performance and self- and proxy observed EF challenges in daily life is not a novel observation in- and outside the ASC research realm (e.g., Davids et al. 2016; Kenworthy et al. 2008; Meltzer et al. 2016; Rabin et al. 2006, 2015; Wallace et al. 2016). In both children and adults with an ASC diagnosis it has been shown that the correlations of the self- and proxy reports with EF task performance is rather low (Kenworthy et al. 2008; Wallace et al. 2016). The BRIEF-A only minimally taps into objective EF as measured by performance-based measures (Meltzer et al. 2016). Ecological validity, however, is relatively strong because the BRIEF-A was designed to tap the complex and multidimensional nature of executive dysfunction as (retrospectively) experienced in daily life. Objective measures, by contrast, typically capture an individual’s best performance under optimal conditions in a laboratory setting (Chaytor and Schmitter-Edgecombe 2003; Sbordone 2000; Wallace et al. 2016). The interpretation of elevated subjective EF scores must account, according to Meltzer et al. (2016), for mood and personality characteristics as important mediators, most notably high anxiety and neuroticism. Adults with an ASC diagnosis have been reported to be high in anxiety and neuroticism (e.g., Kanai et al. 2011; Lever and Geurts 2016b; Schwartzman et al. 2016). This might explain the elevated scores on subjective measurements of ASC individuals, but needs to be tested in future research, but would not directly explain the proxy reports. It is of importance to know what is underlying the experienced EF challenges. Knowing what is causing specific difficulties determines the type of support a person will need in order to reduce their challenges. In psychotherapy contexts, information gained from self-report of EF may provide useful information about patients’ potential ability to engage with treatment strategies such as cognitive restructuring. Also, in older adults with subjective EF dysfunction there could be an opportunity to intervene with training in external or internal strategy use (see Randolph and Chaytor 2013). Such interventions may subtly improve EF and importantly also build confidence and promote a perception of better cognitive functioning, and thus have a particular positive effect on subjective EF. Whether such an intervention is only helpful for those who, next to experiencing EF problems, actually show problems on EF tasks is an open question.

This study has some limitations that are important to keep in mind when interpreting the findings. In order to be able to compare the current findings with the majority of the cognitive aging studies so far, we only included individuals in our ASC group who received an ASC diagnosis later in life (> 55 years of age). The reasons for receiving a late life diagnosis can be diverse (Abbott et al. 2018). It could be due to experiencing less severe symptoms and/or impairments; could be due to better coping and/or compensation skills, or protective environmental factors; and/or this group of older adults recently profited from the clinicians and researchers increased understanding of ASC and better diagnostic tools. Also the sample did not include autistic adults with any intellectual disability or autistic females. This implies that the current findings may not be representative for the whole autistic population. Moreover, all autistic participants visited an outpatient clinic for diagnosis and treatment, which might be caused by the fact that they experienced more severe problems (i.e., mental and physical health problems) than autistic individuals not in treatment. Although the autistic participants received an ASC diagnosis based on extensive diagnostic assessment in which, generally, developmental history is inquired, diagnoses was not verified using internationally known standardized ASC assessment tools. However, there is not yet sufficient evidence that the existing ASC assessment tools are also valid to use in people over 60 years of age (Agelink van Rentergem et al. 2019). Given that the clinic, where the current study is conducted, is known for its autism expertise at old age, we believe that the ASC diagnoses are valid. However, future research may benefit from using standardized methods for diagnosing ASC in older adults. Moreover, while the majority of proxies were partners, not all proxies were similar within and across the included groups of participants. As details regarding the proxy filling out the subjective questionnaire were not recorded sufficiently, we could not test how this might have influenced the proxy reports. A final limitation is that, like each of the previous studies including older participants, the current study had a cross-sectional design. Follow up of these individuals in a longitudinal set up is needed to investigate how cognitive profiles might alter with increasing age.

To conclude, on the objective measures no EF difficulties emerged, but on the subjective measures EF challenges were clearly recognized by the older, and intellectually able, autistic men themselves and by their proxies. Subjective measures offer valuable insight into everyday EF and the experienced problems in an ASC population. In order to know how to support the older autistic men, without an intellectual disability and with a late diagnosis, with their daily life EF challenges, it is important to understand what underlies these EF problems.

## Footnotes

1 Braden et al. (2017) used the Wisconsin Card Sorting Test, while Geurts and Vissers (2012) used a Modified Card Sorting test which is more commonly used in an older population, but this task might have been too easy for the well-educated participants.

2 Please note that in the Lever and Geurts (2016a) paper, separate exploratory analyses were run for those over 50 years of age. Results showed that autistic adults outperformed the comparison group on the visual memory task.

3 Only males were included to increase the homogeneity of the current sample as cognitive profiles are thought to differ between autistic males and autistic females (Lai et al. 2012).

4 Please note that one participants IQ score was 79, but was still included as 80 fell within the reliability interval of this score and there was no reason to assume this person would not be able to perform the other cognitive tasks included.
